# Editorial: Endocrine regulation of homeostasis of water, electrolytes and organic solutes

**DOI:** 10.3389/fendo.2026.1826713

**Published:** 2026-04-07

**Authors:** Alessandro Maria Berton, Emanuele Ferrante

**Affiliations:** 1 Division of Endocrinology, Diabetology and Metabolism, Department of Medical Sciences, University of Turin, Turin, Italy; 2 Endocrinology Unit, Foundation IRCCS Ca’ Granda Ospedale Maggiore Policlinico, Milan, Italy

**Keywords:** acid-base homeostasis, arginine-vasopressin (AVP), chronic kidney disease, diabetes insipidus (DI), extracellular fluid (ECF), hypotonic hyponatremia, syndrome of inappropriate antidiuresis (SIAD), uric acid

The maintenance of water, electrolyte and organic solute balance is a tightly controlled physiological function, ensuring survival. Fluid and solute homeostasis results from the integration of several pathways, including osmoreceptor signaling and vasopressin (AVP) activity, the renin–angiotensin–aldosterone system (RAAS), renal tubular transport and metabolic mediators.

The contributions gathered in this Research Topic collectively illustrate how the disturbances in hydration and solute clearance extend far beyond electrolyte concentration alone.

Daily hydration modulates plasma osmolality (p-Osm) and thereby AVP secretion, inhibiting its release in the case of hypotonicity, while enhancing it during dehydration. Population-based data from the Paracelsus 10,000 study in Salzburg (Austria), reported by Stookey et al., indicate that total water intake exceeding 40 mL/kg/day, including at least 1 L/day of plain water, is associated with more favorable health indicators in community-dwelling middle-aged and older adults.

Notably, even modest chronic elevations in AVP - reflected by copeptin (C terminal-proAVP) - have been associated with insulin resistance, chronic kidney disease (CKD), and increased cardiometabolic risk in various clinical conditions (1). Indeed, persistent activation of V1a receptors (V1aR) may exert deleterious cardiovascular effects by promoting vasoconstriction, platelet aggregation and cardiac remodeling. Moreover, sustained antidiuresis may promote chronic extracellular fluid (ECF) volume expansion, increase cardiac preload, and hemodynamic stress (2). Finally, chronic V2 receptor (V2R) activation has been associated to albuminuria and greater peripheral insulin resistance in non-diabetic CKD patients (3).

A potential interaction between remnant cholesterol and renal function in determining hypertension risk provides further insights into this homeostatic landscape. In their original article Zhao et al., analyzed data from the China Health and Retirement Longitudinal Study (CHARLS), including 5,109 participants with a 9-year follow-up period. They observed a higher prevalence of hypertension among subjects with elevated remnant cholesterol, demonstrating a correlation between increased remnant cholesterol, reduced glomerular filtration rate and hypertension risk. Dyslipidemia contributes to endothelial dysfunction and increased arterial stiffness, while impaired renal perfusion activates RAAS and promotes ECF expansion. Under these premises, hypertension emerges a systemic disorder sustained by dysregulated hormonal signaling and metabolic inflammation (4).

Uric acid handling constitutes an additional component of solute homeostasis with cardiovascular implications (5). In a preclinical murine model, Wu et al. investigated the effects of gigantol, a natural biphenyl compound derived from *Dendrobium officinale*, on serum and urinary uric acid levels, as well as on inflammation pathways associated with hyperuricemia. Gigantol reduced serum uric acid concentrations while increasing its urinary excretion, likely through inhibition of xanthine oxidase activity and restoration of normal urate transporters expression. Furthermore, treatment appeared to reduce hyperuricemia-related inflammatory process. These findings require further confirmations, particularly given the current need for novel anti-gout and hyperuricemia treatments with improved safety and efficacy profiles.

Robust data on cardiovascular outcomes across the full spectrum of AVP-related disorders are currently lacking. Although AVP plays a fundamental role in cardiovascular regulation, as previously discussed, it remains unclear whether chronic deficiency of AVP secretion or, conversely, persistent excess secondary to peripheral insensitivity may influence long-term cardiovascular outcomes.

AVP deficiency (formerly central diabetes insipidus) is a rare and often underrecognized endocrine disorder characterized by impaired synthesis and/or release of AVP from magnocellular hypothalamic neurons, most commonly acquired following neurosurgery (6). Conversely, AVP resistance (previously termed nephrogenic diabetes insipidus) results from peripheral insensitivity to AVP signaling and is frequently iatrogenic (i.e., chronic lithium therapy) (7).

In their review article, Flynn et al. provide updated insights into the diagnosis and management of these conditions, including the differential diagnosis of the polyuria-polydipsia syndrome. Both AVP deficiency and resistance pose clinical challenges, as delayed diagnosis and treatment may lead to dehydration, hypernatremia and substantial morbidity and mortality.

For decades, the water deprivation test followed by desmopressin administration represented the diagnostic gold standard, although it is limited by low accuracy and requires inpatient monitoring. In the recent years, a copeptin-based diagnostic approach has been proposed, enabling a more straightforward and robust diagnostic process, albeit with some limitations that warrant consideration (6, 8, 9). Elevated basal copeptin levels can exclude AVP resistance, while its measurement under appropriate stimulation tests (i.e., hypertonic saline or arginine infusion) allows accurate identification of AVP deficiency.

Nevertheless, ongoing efforts aim to optimize the timely and safe management of hospitalized patients with AVP disorders and to develop shorter, better-tolerated diagnostic protocols that maintain high diagnostic accuracy (10).

On the other hand, hypotonic hyponatremia is the most common electrolyte disorder among hospitalized patients and is independently associated with prolonged length of stay, worse clinical outcomes and increase mortality (11). In most cases ECF volume is preserved, and hyponatremia results from a chronic reduction in free-water clearance leading to dilutional hyponatremia due to the syndrome of inappropriate antidiuresis (SIAD). The underlaying persistent V2R signaling despite hypotonicity may arise from a wide range of diseases, medications and systemic conditions; however, its pathophysiology remains incompletely understood (12, 13). In his minireview Soleimani summarizes current evidence regarding acid-base homeostasis in SIAD-associated hyponatremia. Notably, despite low serum sodium and chloride concentrations, acid–base balance in SIAD is typically near normal, with preserved serum bicarbonate levels, reflecting effective renal compensatory mechanisms. One hypothesis is that an initial dilutional acidosis secondary to ECF expansion may trigger a compensatory renal acid excretion. Additionally, both hypotonicity and V1aR activation have been shown to stimulate renal H^+^-ATPase and, together with enhanced ammoniagenesis, may contribute to the maintenance of systemic acid–base balance.

Beyond its pathogenesis, SIAD still lacks broadly applicable, well tolerated and affordable treatment strategies capable of effectively and sustainably increasing free-water clearance (14–16). In his insightful opinion, Tzoulis critically examines the advantages and limitations of the recently proposed therapeutic approach using empagliflozin in SIAD. Sodium-glucose cotransporter type 2 (SGLT2) inhibitors are oral glucose-lowering agents approved for the treatment of type 2 diabetes mellitus, heart failure and CKD. By inducing persistent glycosuria, they promote osmotic diuresis and are associated with substantial cardiovascular and renal benefits. In the context of SIAD, small randomized controlled trials conducted in both inpatient and outpatient settings have demonstrated a modest increase in serum sodium levels, potentially allowing relaxation of fluid restriction regimens, which are often poorly tolerated (14). Although several aspects remain to be clarified, SGLT2 inhibitors may emerge as an adjunctive therapy to oral urea or adequate dietary protein intake, potentially improving tolerability and adherence in patients with mild-to-moderate hyponatremia, without the cost associated with AVP receptor antagonists.

Taken together, this Research Topic offers valuable examples of contemporary approaches to evaluating the systemic consequences of disrupted regulation of hydro-electrolyte and organic solute homeostasis.
